# Kainic acid hyperphosphorylates tau via inflammasome activation in MAPT transgenic mice

**DOI:** 10.18632/aging.102495

**Published:** 2019-12-02

**Authors:** Xiang-Yu Zheng, Yu-Dan Lv, Feng-Yan Jin, Xiu-Juan Wu, Jie Zhu, Yang Ruan

**Affiliations:** 1 Department of Neurology and Neuroscience Center, The First Hospital of Jilin University, Changchun 130021, China; 2 Department of Hematology, Cancer Center, The First Hospital of Jilin University, Changchun 130021, China; 3 Department of Neurobiology, Care Sciences and Society, Karolinska Institute, Stockholm 141 86, Sweden; 4 Key Laboratory of Pathobiology, Ministry of Education, College of Basic Medical Sciences, Jilin University, Changchun 130021, China

**Keywords:** kainic acid, NLRP3, NF-κB, interleukin-1β, tau

## Abstract

The excitotoxicity induced by kainic acid (KA) is thought to contribute to the development of Alzheimer’s disease (AD); however, the mechanisms underlying this excitotoxicity remain unknown. In the current study, we investigated the dynamic changes in tau phosphorylation and their associations with the excitotoxicity induced by intraperitoneal injection of KA in the mouse brain. We found that KA-induced excitotoxicity led to sustained hyperphosphorylation of tau in MAPT transgenic (Tg) mice. By using cultured microglia and mouse brains, we showed that KA treatment specifically induced endoplasmic reticulum (ER) stress, which was characterized by activation of the major biomarkers of ER, such as ATF6, GRP78, and IRE1, and resulted in stimulation of inflammasomes. KA receptors (KARs), such as Girk1, were determined to be involved in this KA-induced ER stress. ER stress was also shown to activate inflammasomes by stimulating the expression of the two major components of inflammasomes, nucleotide binding oligomerization domain (NOD)-like receptor (NLR) protein 3 (NLRP3) and nuclear factor (NF)-κB, and eventually causing the production of interleukin-1β (IL-1β). Inhibition of NLRP3 or NF-κB by Bay11-7082 resulted in reduction of KA-induced IL-1β production. Our results also revealed the positive effects of IL-1β on tau phosphorylation, which was blocked by Bay11-7082. Notably, the results indicate that Bay11-7082 acts against KA-induced neuronal degeneration, tau phosphorylation, and memory defects via inflammasomes, which further highlight the protective role of Bay11-7082 in KA-induced neuronal defects.

## INTRODUCTION

Alzheimer’s disease (AD), also named as dementia is pathologically characterized by multiple factors, such as the presence of amyloid β-protein (Aβ) and tau in the brain, especially in the hippocampus [1, 2]. Although treatments to prevent the progression of AD and reverse its effects have not yet been developed, recent studies have revealed much about the genetic mechanisms underlying such diseases. For instance, the study by Heneka et al. [3] suggested that the nucleotide binding oligomerization domain (NOD)-like receptor (NLR) protein 3 (NLRP3) inflammasome plays a role in AD by demonstrating increased caspase-1 expression levels in brains with AD. Accordingly, several complexes of inflammasomes have been identified, each of which is named after its NLRP that exerts the damaging biological functions [4]. In addition, the formation of inflammasome will recruit the inflammatory enzyme, caspase-1 [5]. The activation of caspase-1 results in secreting pro-inflammatory cytokines including interleukin (IL)-1β, which is responsible for inducing a series of immunoresponses. In detail, recruitment of procaspase-1 to inflammasome will induce the oligomerization and autocatalysis of caspase-1, leading to the release of active form of caspase-1 fragments and IL-1β. More importantly, IL-1β has the ability to phosphorylate tau, which result in worsening AD [6], leading to impair learning and memory in AD mice [7, 8]. Moreover, deactivation of IL-1β *in vivo* protects AD animals from the risk of the disease [9]. Overall, the abovementioned mechanisms might potentially collaboratively contribute to the roles of the NLRP3 inflammasome in behavioral changes and cognitive deficiencies associated with AD.

Although the mechanism underlying NLRP3 activation remains unclear, several upstream regulations have been suggested, such as the generation of ion fluxes, phagosomal destabilization mitochondrial, reactive oxygen species (ROS) or release of lysosomal cathepsins. Specifically, in macrophages and monocytes, NLRP3 activation is always accompanied by the production of ROS, which indicates that mitochondrial ROS accounts for the activation of NLRP3 [10–12]; moreover, K^+^ fluxes have been implicated in NLRP3 activation [4]. Concurrently, NF-κB mediates the up-regulation of NLRP3 and proIL-1β transcripts in response to ROS stimulation [10]. Further mechanistic investigations have also revealed the key roles of NF-κB in driving the transcription of NLRP3 by stimulating the activity of Toll-like receptor (TLR) or with NLR ligands [10]. In addition to these mechanisms, endoplasmic reticulum (ER) stress was recently identified to activate NF-κB in several experimental models [13–15], which is probably associated with the activity of NLRP3. These reports also indicated the possible involvement of ER stress in activating inflammasomes and subsequently exacerbating AD. ER stress has been actually accepted to be associated with the early events in and progression of AD [16]. Moreover, the neurons of AD patients showed abundant levels of the biomarker of ER stress, GRP78, and ERK phosphorylation [17, 18]. More interestingly, ER stress can activate the NLRP3 inflammasome [19, 20]. These reports indicate that ER stress might potentially exacerbate AD via inflammasome activation.

Glutamate receptors have been recently reported to be activated by kainic acid (KA), which are responsible for inducing ER stress [21]. In addition, salubrinal, an ER stress inhibitor treatment suppressed neuron death in KA-stimulated hippocampus [22], indicating that KA can induce biological functions via activating ER stress. Similarly, melatonin has been shown to mitigate KA-induced neuronal death by alleviating ER stress in neuroblastoma (N)2a cells [23], and ER stress is known to mediate the KA-induced the phosphorylation of tau in the hippocampus-derived neurons [24]. In line with these previous studies, we current show that KA induces the phosphorylation of tau via the ER-activated inflammasome pathway in the current investigation. Inhibition of inflammasome activation attenuates the excitotoxicity of neurons via alleviating ER stress in KA-activated experimental models.

## RESULTS

### Kainic acid treatment activates inflammasome and induces tau phosphorylation in the brains of MAPT Tg mice

**Figure 1:**
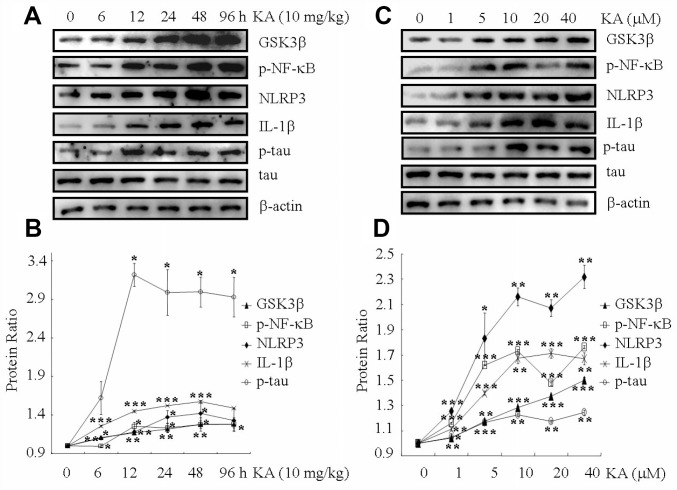
**KA augments inflammasome activity and tau phosphorylation in vivo and in vitro.** (**A**, **B**) Truncation of GSK3β, phosphorylation levels of NF-κB, and the expression levels of NLRP3 and IL-1β as well as the phosphorylation of tau in the KA-treated mouse brain at different time points. (**C**, **D**) Truncation of GSK3β, phosphorylation levels of NF-κB, and the expression levels of NLRP3 and IL-1β as well as the phosphorylation of tau in KA-treated mixed cells. The optical density of bands in western blots was analyzed by Image J software (*P < 0.05, **P < 0.01, ***P < 0.001 vs. controls; the significant differences from the respective values were determined by one-way analysis of variance test. N = 3 for western blotting).

KA is widely considered to be responsible for inducing status epileptics. Besides, KA is also reported to impair leaning ability and memory, which result in neurodegeneration [26]. To verify the toxicity of KA in neurons, 10 mg/kg of KA were intraperitoneally injected to MAPT Tg mice, which were then measured GSK3β truncation, NF-κB phosphorylation, NLRP3, ASC and IL-1β, as well as tau phosphorylation in the mice brains at 6, 12, 24, 48, 96 h. At the indicated time points after treatment with KA, GSK3βtruncation, NF-κB phosphorylation, NLRP3 and IL-1β expression, as well as tau phosphorylation were significantly elevated in the brains of the MAPT Tg mice (Figure 1A, 1B and Supplementary Figure 1A, 1B). Of note, the upregulation of these molecules reached plateau after 12 h treatment. 96 h after treatment with KA, the levels of GSK3βtruncation, NF-κB phosphorylation, NLRP3 and IL-1β expression, as well as tau phosphorylation were decreased to a small extent (Figure 1A, 1B). Of note, the phosphorylated NF-κB has the ability to enter the nucleus of the cells (Supplementary Figure 2A, 2B). Therefore, we harvested KA-treated hippocampus at 48 h for the following experiments.

We further assayed GSK3βtruncation, NF-κB phosphorylation, NLRP3 and IL-1βexpression, as well as tau phosphorylation in the cell mixture (50% N2a and 50% BV2). At 48 h, the truncation of GSK3β and the phosphorylation of NF-κB increased significantly in the cell mixture, and the phosphorylation of NF-κB reached the maximum plateau at 10 μM KA treatment (Figure 1C, 1D). Accordingly, the phosphorylated NF-κB has the ability to enter the nucleus of the cells (Supplementary Figure 2C, 2D). In addition, the expression of NLRP3, ASC and IL-1β was elevated in KA (10 μM)-treated BV2 cells (Figures 1C, 1D and Supplementary Figure 1C, 1D). More importantly, tau phosphorylation also reached the plateau in KA-treated mixed cells (Figure 1C, 1D). Considering the neurotoxicity of KA treatment, we chose to use 10 μM KA in our subsequent experiments. Collectively, our results implied that KA treatment could efficiently increase the truncation of GSK3β, phosphorylation of NF-κB, expression of NLRP3 and IL-1β as well as the phosphorylation of tau both *in vivo* and *in vitro*.

### NF-κB and NLRP3 are highly expressed in the brains of MAPT Tg mice

**Figure 2:**
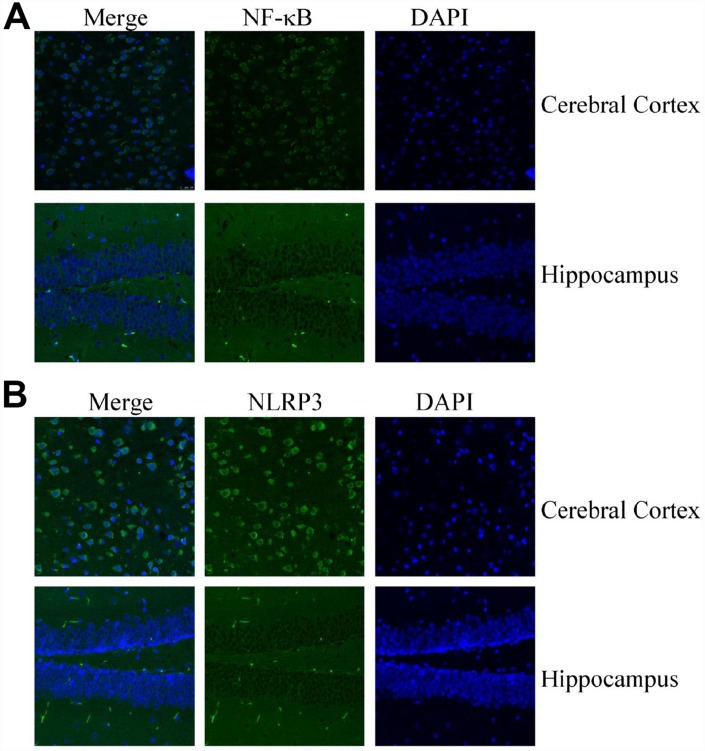
**Confocal fluorescence microscopy shows the expression of NF-κB and NLRP3 in the brains of MAPT Tg mice.** Images from a single z-plane of the brains of MAPT Tg mice at 6 months of age by using antibodies against NF-κB (**A**) and NLRP3 (green) (**B**) and nucleus (blue DAPI stain). Scale bar = 25 μm.

In light of the critical roles of inflammasomes in AD [3], we further measured the expression of NF-κB and NLRP3 in MAPT Tg mice. Confocal microscopy was used to study the distribution of NF-κB and NLRP3 in 6-month-old MAPT Tg mice (Figure 2). The findings showed that the expressing levels of NF-κB are highly elevated in the brains of MAPT Tg mice (Figure 2A). Similarly, NLRP3 was also highly expressed in the cerebral cortex and hippocampus of 6-month-old MAPT Tg mice (Figure 2B). Of note, the expression levels of NF-kB and NLRP3 in the cerebral cortex were higher than those in the hippocampus (Figure 2A, 2B). The data from confocal microscopy clearly illustrated that NF-κB and NLRP3 were highly expressed in the brains of MAPT Tg mice. Moreover, the findings also indicated that NF-κB and NLRP3 might potentially contribute to KA-induced tau phosphorylation.

### Grik1 mediates KA-induced ER stress

**Figure 3:**
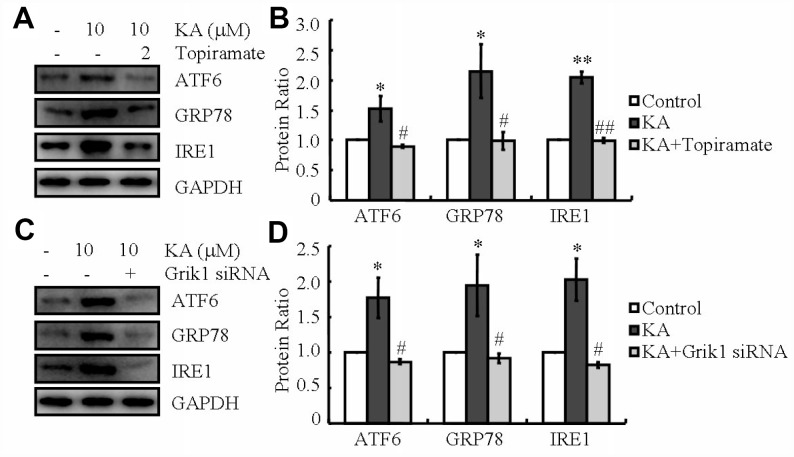
**Grik1 mediates KA-induced ER stress.** (**A**, **B**) N2a cells were treated with 10 μM KA in the absence or presence of 2 μM topiramate for 48 h. (**C**, **D**) Grik1 siRNA (200 ng) was transfected to N2a cells before treatment with KA (10 mg/kg). The protein levels of ATF6, GRP78, and IRE1 were determined by western blots. The optical density of bands in western blots was analyzed by Image J software (*P < 0.05 vs. the control group; #*P* < 0.05; ##*P* < 0.01 vs. the KA-only treatment).

KA receptors (KARs) are encoded by a series of individual genes, such as GluR5-7, KA-1 and KA-2. These receptors are widely distributed in the brain, whose expression is associated with cell death [27]. To determine if KARs mediate the effects of KA on AD, siRNA interference experiments were performed to identify the roles of KARs in the phosphorylating tau in the cell mixture (data not shown). The results revealed that the phosphorylation of tau was enhanced in KA (10 μM)-treated cells compared to that of control group. In addition, phosphorylation of tau was obviously attenuated in KA-treated Grik1 knockdown cells than in KA-treated cells (data not shown). As previously reported in relation to KA-induced phosphorylation of tau via ER stress [24], we firstly determined the ability of KA to regulate ER stress via Grik1. To achieve this purpose, we treated N2a cells with the antagonist of Grik1, topiramate (2 μM), together with KA (10 μM) for 48 h. Consistently, the levels of ATF6, GRP78, and IRE1 rose in KA-treated N2a cells (Figure 3A, 3B). By further incubated with topiramate, the expression of ATF6, GRP78, and IRE1 significantly decreased (Figure 3A, 3B), indicating that Grik1 mediated the KA-induced ER stress.

To exclude the potential non-specificity of the Grik1 antagonist, Grik1 siRNA was employed to transfect the N2a cells. As shown in the western blots, Grik1 knockdown blocked the positive effects of KA on the protein production of ATF6, GRP78, and IRE1 in N2a cells (Figure 3C, 3D). All in all, these data revealed that Grik1 mediates the effects of KA on activating ER stress.

### KA-induced ER stress promotes the activities of the inflammasome and results in the phosphorylation of tau

**Figure 4:**
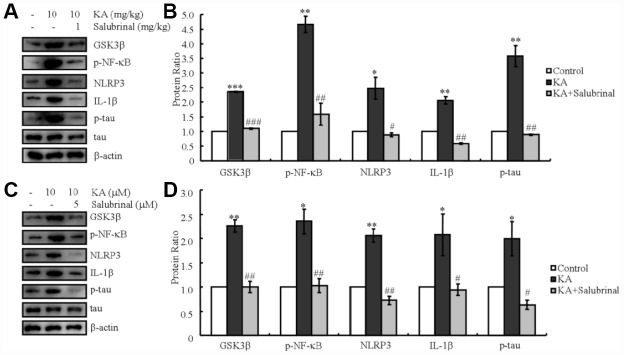
KA-induced tau hyperphosphorylation is highly correlated with ER stress-activated inflammasome. (**A**, **B**) Truncation of GSK3β, phosphorylation levels of NF-κB, and the expression levels of NLRP3 and IL-1β as well as the phosphorylation of tau were determined by western blots of samples from MAPT Tg mice treated with KA (10 mg/kg) and/or salubrinal (1 mg/kg) together with KA (10 mg/kg). The KA group was given i.p. injection of 10 mg/kg KA. The salubrinal+KA group mice were additionally given i.p. injections of 1 mg/kg Bay11-7082. Both groups were assessed after 48 h. (**C**, **D**) Truncation of GSK3β, phosphorylation levels of NF-κB, and the expression levels of NLRP3 and IL-1β as well as the phosphorylation of tau were determined by western blots in cells treated with KA (10 μM) and/or salubrinal (5 μM) together with KA (10 μM). The KA group was treated with 10 μM KA. The salubrinal+KA group was additionally treated with 5 μM salubrinal. Both groups were assessed after 48 h. The optical density of the bands in western blots was analyzed by Image J software (*P < 0.05; **P < 0.01; ***P < 0.001 vs. the control group; #*P* < 0.05; ##*P* < 0.01; ###*P* < 0.001 vs. the KA-only group).

Since a previous report stated that ER stress could activate the NLRP3 inflammasome [19, 20], we determined the ability of KA to regulate the activity of the inflammasome via ER stress. As expected, the levels of GSK3β truncation, NF-κB phosphorylation, and NLRP3 and IL-1β expression increased in KA-treated mice (Figure 4A, 4B). In addition, tau phosphorylation was also induced by KA treatment (Figure 4A, 4B). Then, we treated mice with the inhibitor of ER stress, salubrinal (1 mg/kg) together with KA (10 mg/kg) for 48 h. Treatment with salubrinal, the GSK3β truncation, NF-κB phosphorylation, NLRP3 and IL-1β expression, as well as tau phosphorylation significantly decreased (Figure 4A, 4B), which indicates that KA addition can efficiently activate ER stress by promoting the activities of the inflammasome and eventually result in the phosphorylation of tau *in vivo*.

In order to further validate the effects of ER stress on AD *in vivo*, a mixture of cells was treated with KA (10 μM) in the absence or presence of salubrinal (5 μM) for 48 h. In parallel with the data obtained *in vivo*, KA treatment induced GSK3β truncation, NF-κB phosphorylation, NLRP3 and IL-1β expression, as well as tau phosphorylation (Figure 4C, 4D). With the addition of salubrinal, the GSK3β truncation, NF-κB phosphorylation, NLRP3 and IL-1β expression, as well as tau phosphorylation significantly decreased (Figure 4C, 4D), which confirms the critical effects of ER stress on mediating the KA-induced activities of the inflammasome and leading to the phosphorylation of tau *in vivo*.

### GSK3β mediates the ER stress-induced inflammasome activity and tau hyperphosphorylation

**Figure 5:**
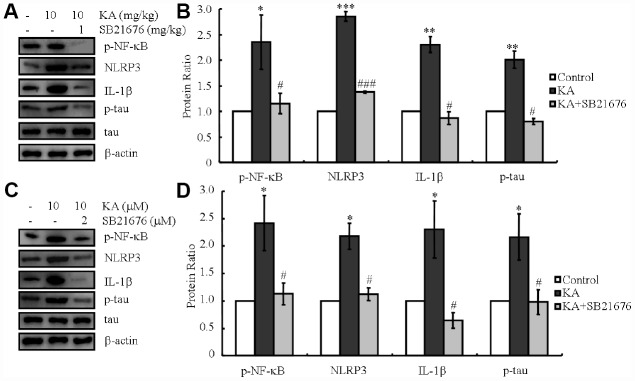
**Inhibitor of GSK3β, SB216763, attenuates the phosphorylation of NF-κB, expression of NLRP3 and IL-1β, as well as the phosphorylation of tau.** (**A**, **B**) Phosphorylation levels of NF-κB and the expression levels of NLRP3 and IL-1β as well as the phosphorylation of tau were determined in western blots of samples from MAPT Tg mice treated with KA (10 mg/kg) and/or SB216763 (1 mg/kg) together with KA (10 mg/kg). The KA group was given i.p. injection of 10 mg/kg KA. The SB216763+KA group mice were additionally given i.p. injections of 1 mg/kg SB216763. Both groups were assessed after 48 h. (**C**, **D**) Phosphorylation levels of NF-κB and the expression levels of NLRP3 and IL-1β as well as the phosphorylation of tau were determined by western blots in cells treated with KA (10 μM) and/or SB216763 (2 μM) together with KA (10 μM). The KA group was treated with 10 μM KA. The SB216763+KA group was additionally treated with 2 μM SB216763. Both groups were assessed after 48 h. The optical density of bands in western blots was analyzed by Image J software (*P < 0.05; **P < 0.01; ***P < 0.001 vs. the control group; #*P* < 0.05; ###*P* < 0.001 vs. the KA-only group).

ER stress-dependent inflammasome activation led to the elevation of NLRP3 and IL-1β levels, which promoted the phosphorylation of tau (Figure 4). ER stress also stimulated the truncation of GSK3β *in vitro* and *in vivo* (Figure 4). Both truncation of GSK3β and activation of inflammasome boosted the phosphorylation of tau [3, 24, 28, 29]. The upstream and downstream relationship between GSK3β and inflammasome in regulating tau phosphorylation has not been resolved. Accordingly, we injected KA (10 mg/kg) intraperitoneally in MAPT Tg mice with or without the GSK3β inhibitor, SB216763 (1 mg/kg), and observed the findings after 48 h. As shown in the western blots, KA treatment prominently increased the NF-κB phosphorylation, NLRP3 expression and IL-1β as well as the phosphorylation of tau, whereas treatment with the GSK3β inhibitor SB216763 reduced the respective levels (Figure 5A, 5B)

In order to further validate the data *in vivo*, a cell mixture was treated with KA (10 μM) in the absence or presence of SB216763 (2 μM) for 48 h. In parallel with the data obtained *in vivo*, KA treatment induced NF-κB phosphorylation, NLRP3 and IL-1βexpression as well as tau phosphorylation (Figure 5C, 5D). With the addition of SB216763, the NF-κB phosphorylation, NLRP3 and IL-1β expression, as well as tau phosphorylation significantly decreased (Figure 4C, 4D), suggesting that GSK3β plays a key role in mediating ER stress-induced inflammasome activities and causing tau phosphorylation *in vivo*.

### Inflammasome is responsible for the expression of IL-1β, leading to the phosphorylation of tau

**Figure 6:**
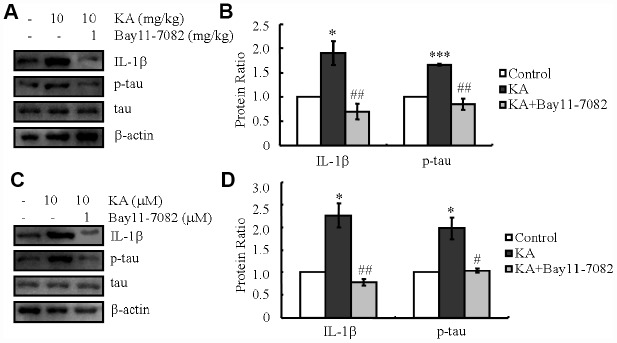
**KA-induced activation of inflammasome promotes the phosphorylation of tau.** (**A**, **B**) The expression levels of IL-1β and the phosphorylation of tau were determined by western blots of samples from MAPT Tg mice treated with KA (10 mg/kg) and/or Bay11-7082 (1 mg/kg) together with KA (10 mg/kg). The KA group was given i.p. injection of 10 mg/kg KA. The Bay11-7082+KA group mice were additionally given i.p. injections of 1 mg/kg Bay11-7082. Both groups were assessed after 48 h. (**C**, **D**) The expression levels of IL-1β and the phosphorylation of tau were determined by western blots using cells treated with KA (10 μM) and/or Bay11-7082 (1 μM) together with KA (10 μM). The KA group was treated with 10 μM KA. The Bay11-7082+KA group was additionally treated with 1 μM Bay11-7082. Both groups were assessed after 48 h. The optical density of bands in western blots was analyzed by Image J software (*P < 0.05; ***P < 0.001 vs. the control group; #*P* < 0.05; ##*P* < 0.01 vs. the KA-only group).

NF-κB has been reported to activate the NLRP3 inflammasome [30]. In agreement with our study, the NF-κB phosphorylation, NLRP3 and IL-1β expression rose in KA-stimulated mice, which reached the maximum plateau at 48 h (Figure 1A, 1B). We therefore treated the mice with KA (10 mg/kg) without or with an inhibitor of inflammasome [31, 32], Bay11-7082 (1 mg/kg). After 48 h, the phosphorylation of tau and the expression levels of IL-1β significantly decreased with the addition of Bay11-7082 (Figure 6A, 6B), suggesting the key roles of KA in inducing the expression of IL-1β via inflammasome activation. In order to further validate the critical roles of the inflammasome in the expression of IL-1β and the phosphorylation of tau, we deployed Bay11-7082 (1 μM) in KA (10 μM)-treated mixed cells. We found that Bay11-7082 inhibited the expression of IL-1β (Figure 6C, 6D) and decreased the phosphorylation of tau in KA-treated cells (Figure 6C, 6D), suggesting the critical role of the inflammasome in regulating the expression of IL-1β and tau phosphorylation.

### KA upregulates tau phosphorylation by inducing the expression of IL-1β

**Figure 7:**
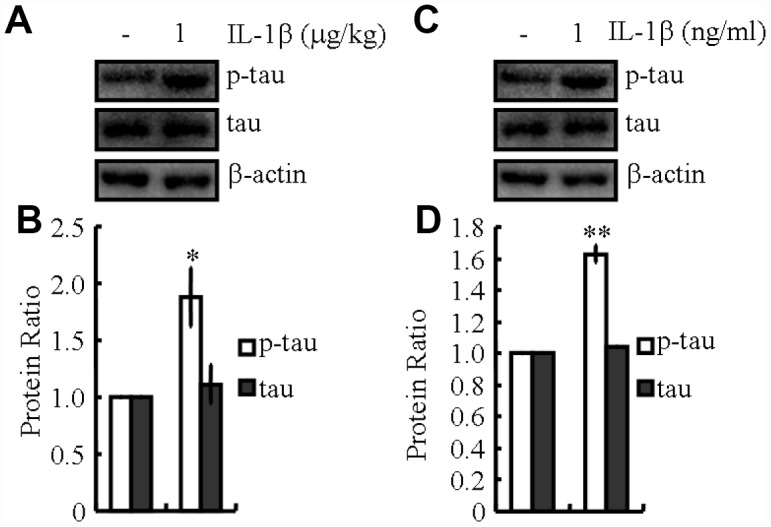
**IL-1β mediates inflammasome-induced tau phosphorylation.** (**A**, **B**) The phosphorylation levels of tau were determined by western blots in IL-1β (1 μg/kg)-treated MAPT Tg mice. The IL-1β group was given i.p. injection of 1 μg/kg IL-1β and assessed after 48 h. (**C**, **D**) The phosphorylation levels of tau were determined by western blots in IL-1β (1 ng/ml)-treated N2a cells. The IL-1β group was treated with 1 ng/ml IL-1β and assessed after 48 h. The optical density of bands in western blots was analyzed by Image J software (*P < 0.05; **P<0.01 vs. the control group).

As previously shown, our observations suggested the essential effects of KA on activating the inflammasome (Figure 1). Accordingly, we speculated that KA potentially contributes to abnormal tau phosphorylation. In line with the changes in inflammasome biomarkers, tau phosphorylation was up-regulated significantly in KA-treated mice (Figure 1), suggesting the critical roles of KA-induced inflammasome in modulating tau phosphorylation. To verify this hypothesis, mice or cells were treated with Bay11-7082 and KA. The results revealed that Bay11-7082 obviously blocked the induction of tau phosphorylation in KA-activated mice (Figure 6), suggesting that the inflammasome could act upstream of IL-1β expression and abnormal tau phosphorylation. To further assay the requirement of IL-1β in KA-induced tau phosphorylation, we treated MAPT Tg mice with IL-1β (1 μg/kg) and found that IL-1β induced the phosphorylation of tau *in vitro* and *in vivo* (Figure 7). Together with our biochemical results, these findings demonstrated that inflammasome-activated IL-1β could mediate KA-stimulated tau phosphorylation.

### KA induces the cognitive decline of APP23 mice via activating inflammasome

**Figure 8:**
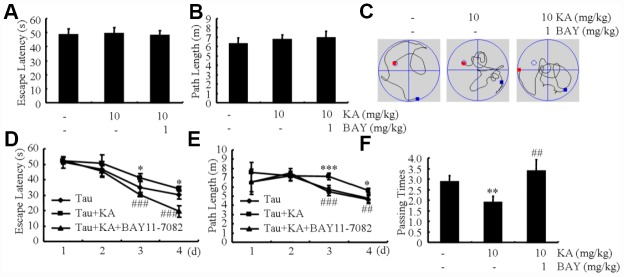
**Bay11-7082 mitigates KA-induced memory deficits in the Morris water maze test.** (**A**, **B**) During the first 2 days of visible platform tests, the KA and Bay11-7082 treated and control MAPT Tg mice exhibited a similar latency to escape onto the visible platform. *P* > 0.05 with Student’s t-test. (**C**, **D**) In the hidden platform tests, KA-treated MAPT mice showed a longer latency and length to escape onto the hidden platform on the 3^rd^ and 4^th^ days, which was ameliorated by the addition of Bay11-7082 on the 4^th^ day. *P < 0.05; ***P < 0.001 vs. the control group; ##*P* < 0.01; ###*P* < 0.001 vs. the KA-treated group by ANOVA. (**E**) In the probe trial on the 7^th^ day, the KA-treated MAPT Tg mice traveled into the third quadrant, where the hidden platform was previously placed, significantly less times than controls, which was improved by the treatment with Bay11-7082. **P < 0.01 vs. the control group; ## *P* < 0.01 vs. the KA-treated group by ANOVA.

**Figure 9:**
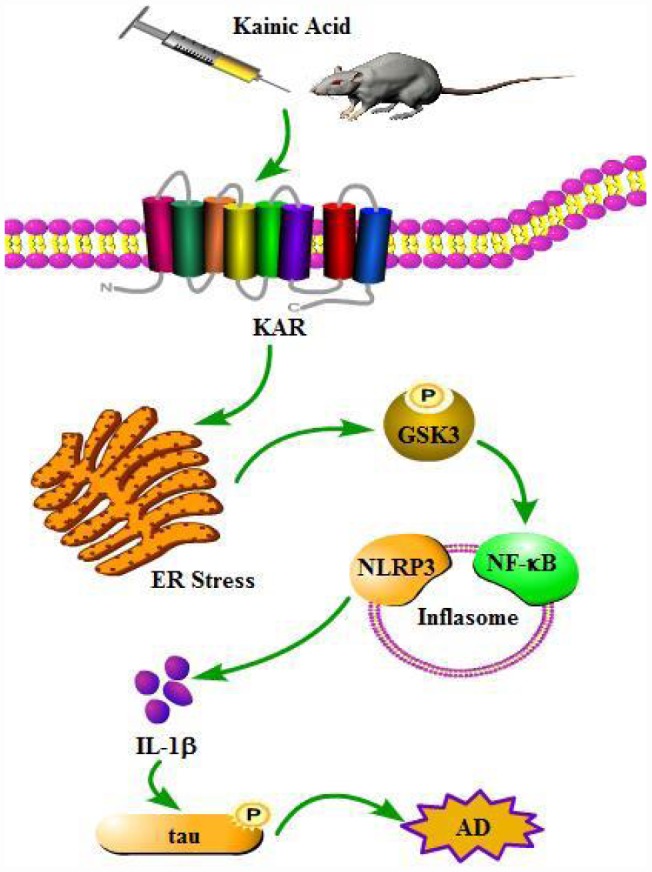
**A functional model of KA-induced inflammasome activity through tau phosphorylation and memory deficits were exacerbated in an ER stress-dependent mechanism.** KA treatment triggers activation of the inflammasome and causes the phosphorylation of NF-κB, leading to NLRP3 upregulation via ER stress. Upregulated NLRP3 eventually results in the phosphorylation of tau by enhancing the expression of IL-1β. Bay11-7082 inhibits KA-induced IL-1β activation and tau phosphorylation by alleviating the activity of inflammasome, which ultimately improved the cognitive decline in MAPT Tg mice.

Since the loss of memory is closely related with hyperphosphorylation of tau [33], we speculated that dephosphorylation of tau by inhibiting inflammasome activity will indicate the beneficial effect of Bay11-7082 on AD. To judge if Bay11-7082 has the ability to attenuate the loss of memory by KA, MWM test was performed to assay the cognitive impairment of the mice. The mice displayed similar tendency to the visible platform within the first 2 d of tests, suggesting no defective diseases of the mice (Figure 8A, 8B). For the following experiments, we found that the mice took longer time to find the platform in KA-treated group compared to that of vehicle-treated controls (Figure 8C). Furthermore, Bay11-7082 treatment partially blocked the effects of KA on increasing the escape latency of KA-treated mice (Figure 8C). In agreement with escape latency, the mice took shorter path length to find the platform in KA+Bay11-7082 group than that of the KA-treated controls (Figure 8D). On the 7 d, the platform was removed from the MWM. The mice showed more times of crossing platform in the KA+Bay11-7082 group than that of the KA-only controls (Figure 8E). Taken together, these results revealed the mechanisms underlying the effects of KA in exacerbating AD via inflammasome activation (Figure 9).

## DISCUSSION

Excessive excitotoxicity has been reported to contribute to neurodegenerative processes [34]. Glutamate and the associated excitatory amino acids can impair neurons by inducing apoptosis *in vitro* and *in vivo* [35, 36]. As an analogue of glutamate-associated amino acids, KA was recently reported to be associated with AD, especially with tau hyperphosphorylation [24]. In line with the previous investigation, we revealed that KA has the ability to activate ER stress and GSK3β. In addition, we showed that ER stress mediated the effects of KA on activating the inflammasome. Moreover, GSK3βinhibition resulted in inflammasome deactivation, which suggests that GSK3β could occur upstream of the inflammasome to regulate the processes underlying AD. Importantly, we observed that Bay11-7082 partially blocked the KA-induced tau hyperphosphorylation and cognition disabilities by suppressing inflammasome activity. Meanwhile, we also noticed that the inflammasome might be a potential therapeutic target for AD (Figure 9).

KA was first shown to induce seizures. Further studies suggested that KA has the ability to induce neurodegeneration by intraperitoneal administration [37, 38]. It binds to specific excitatory amino-acid receptors, such as Grik1-3, KA1, and KA2 in the central nervous system (CNS) [39]. For instance, the administration of KA agonist will result in the death of neurons by influencing the activity of the AMAP, a kainate class of glutamate receptors [40–42]. In rodents, treatment with KA induced recurrent seizures, behavioral changes, and neurodegeneration in the brain [43, 44]. More specifically, the excitotoxicity mediated by the above receptors may underlie the pathogenesis of neurodegenerative diseases, such as Alzheimer’s disease (AD). Likewise, we found that KA exacerbates AD via hyperphosphorylation of tau in the brains of MAPT Tg mice. Notably, KA has the ability to induce the phosphorylation of tau and death of neurons through activation of the GSK3β and CDK5 pathways *in vivo* and *in vitro* [45, 46]. Activation of GSK3β and CDK5 are closely associated with the hyperphosphorylation of tau in AD. The abnormally hyperphosphorylated tau will deposit in intracellular neurofibrillary tangles (NFTs), which related to impair memory in AD patients [33]. Consistently, our data also showed that KA induced cognitive decline in MAPT Tg mice (Figure 8).

Mechanistically, ER stress was found to be involved in regulating tau phosphorylation in KA-treated mice (Figure 4). Actually, various disorders are induced by ER stress in the CNS [47]. Moreover, constant illumination was also reported to result in ER damage, which up-regulated tau hyperphosphorylation and memory impairment [48]. These investigations indicated that ER stress is involved in regulating the process of AD. Moreover, it has been reported that the hyperphosphorylation of tau and the activation of GSK3β were down-regulated by suppressing the activity of of ER stress [24]. Truncation of c-terminal GSK3β is also related to activate calpain [29], which is responsible for cleaving CDK5/p35 to CDK5/p25 [28]. The latter factor is necessary for tau phosphorylation in AD brains [28]. Hence, ER stress mediates KA-induced tau phosphorylation in MAPT Tg mice.

More intriguingly, ER stress was identified to induce tau phosphorylation via activation of the inflammasome (Figure 4). Indeed, ER stress has been found to activate NF-κB in several experimental models [13–15], which potentially contributes to the activation of NLRP3. Moreover, ER stress has been accepted to be associated with the early events in AD and the progression of the disease [16]. Furthermore, the biomarker of ER stress, GRP78, and the phosphorylation of ERK were identified to be highly expressed in neurons of AD patients [17, 18]. More closely, ER stress has the ability to activate NLRP3 inflammasome [19, 20]. Together with our study, all these reports indicate that ER stress might potentially exacerbate AD via inflammasome activation.

It is worth to mention that NLRP3 was activated in the microglia cells around β-amyloid plaques (APs) [3], suggesting the key roles of microglial activation of the NLRP3 inflammasome for the pathogenesis of AD. In fact, neuroinflammation is always accompanying the pathogenesis of AD. For example, neurodegeneration is reported to be regulated by deregulating and alternating the activity of microglials in AD [49]. The over-loading of proinflammatory chemokines from microglials probably exert biological functions on neurons and eventually lead to impair brains [50]. More specifically, IL-1β has shown its effects on worsening AD via phosphorylating tau [6], which results in impairment of learning and memory in AD mice [7, 8]. Additionally, inhibiting the activity of IL-1β *in vivo* protected the AD animal from the risk of the diseases [9]. In view of these observations, the current study further reveals that inflammasome promoted the production and secretion of IL-1β, which contribute to the phosphorylation of tau. Noteworthy, the production and secretion of IL-1β is ascribed to the activity of NLRP3 inflammasome. Thus, considering the paucity of relevant data, pyroptosis in AD should be further studied.

Finally, tau has the ability to stabilize and assemble the microtubules [51]. However, hyperphosphorylation of tau will lose its biological function on stabilize microtubules, which result in AD [52]. As phosphorylated tau is the main component of neurofibrillary tangles (NFTs) in AD [53], its deposition will impair the memory and cognition of AD patients [33]. Hence, revealing the inherent mechanisms for regulating the phosphorylation of tau will provide appropriate strategies to treat AD. For this reason, our experiments were delicately designed, and as expected, we found that the inhibitor of NLRP3 inflammasome, Bay11-7082 attenuated the activating effects of KA on the phosphorylation of tau, and the impairment of memory in MAPT Tg mice (Figure 9).

## MATERIALS AND METHODS

### Mice and treatment protocol

MAPT [B6;C3-Tg(Prnp-MAPT*P301S)PS19Vle/J] mice were obtained from The Jackson Laboratory (Stock #008169). The MAPT mouse line is transgenic for the human MAPT protein carrying the P301S mutation under the direction of the mouse prion protein promoter. The expression of MAPT^P301S^ protein is 5-fold higher than that of endogenous mouse MAPT protein. Insoluble hyperphosphorylated MAPT^P301S^ protein accumulates in the brain with age, which caused decreasing microtubule binding/density. At 9 months of age, significant neuron degeneration in the hippocampus occurs, though this phenomenon has been found as early as 5-month-old. Moreover, defective translocation of ER proteins in affected neurons is observed as early as 3 months of age. All animal procedures were approved by the Institutional Animal Care and Use Committee of Jilin University, which was in compliance with the Guidelines for the Care and Use of Laboratory Animals of the U.S. National Health Institute. The mice were housed five per cage in a room maintained at 22 ± 2°C with an alternating 12-h light-dark cycle. Food and water were available *ad libitum*.

On the basis of a previous study [25], a total of 6 mice were treated with an intraperitoneal (i.p.) injection of 10 mg/kg KA (Sigma-Aldrich Corp, St. Louis, MO, USA) emulsified in 0.9% PBS (-). To validate the induction of KA neurotoxicity, behavioral observations were made every 30 min for 4 h after KA injection. The mice that did not display general limbic seizure activity within 90 min after the KA injection were excluded from further study. Mice in the control group (n = 6) were injected with PBS (-). Animals in the experimental group were sacrificed 6, 12, 24, 48, 96 h after KA treatment.

In the short-term treatment assessments, mice were randomly divided into three groups: KA-only group (10 mg/kg); salubrinal (10 mg/kg) (Sigma-Aldrich Corp, St. Louis, MO, USA), SB216763 (10 mg/kg) (Tocris Bioscience, Bristol, UK), or Bay11-7082 (1 mg/kg) (Sigma-Aldrich Corp, St. Louis, MO, USA) administration together with KA (10 mg/kg); and vehicle-treated control group (Control). In the long-term treatment assessments, 3-month-old mice were similarly treated with KA (10 mg/kg) and Bay11-7082 (1 mg/kg)/KA per day for 3 months before the Morris water maze (MWM) test and brain tissue collection. All mice were sacrificed after treatment, and their brain tissue was harvested for further tests.

### Morris water maze test

To assess cognitive changes, the Morris water maze (MWM) test was conducted in a circular water tank (1.4 m in diameter and 40 cm in height) filled with water to the depth of 20 cm and maintained at 21 ± 1°C. The tank was divided into four equal quadrants. A submerged square platform was placed in the third quadrant of the tank with its top surface 1 cm below the water surface. The mice were placed in the pool at four possible start locations facing the wall of the pool, and a camera was simultaneously activated. Each mouse was allowed up to 60 s to locate the platform. The trial was terminated when the mouse found the platform within 60 s. If a mouse failed to find the platform within 60 s, it was guided by a researcher to locate the platform and allowed to stay there for 2-3 s. Each mouse was conditioned three times per day for 2 days to allow adaptation to the pool environment (visible platform training) and then tested three times per day for 4 days to find the hidden platform (hidden platform training). The latency (the time taken to locate the platform in water), distance, and swim speed were recorded using an automated video tracking software package (NoldusEtho Vision 2.3.19, Netherlands). On the seventh day, the platform was removed and the number of times the mouse passed the original location of the platform was tracked and recorded by video.

### Immunofluorescent staining

Frozen sections (thickness, 10 μm) of mice were blocked with 5% normal goat serum in tris-buffered saline (TBS) for 30 min, followed by incubation with primary antibodies overnight at 4°C in TBS containing 5% goat serum and 0.1% Triton. Primary antibodies used included rabbit monoclonal NF-κB (1:50, Cell Signaling Technology, Danvers, MA, USA) and rabbit monoclonal NLRP3 (1:100, Cell Signaling Technology, Danvers, MA, USA). After washing with TBS, the sections were incubated with anti-Mouse IgG (H+L), F(ab')_2_ Fragment (Alexa Fluor^®^488 Conjugate) (1:1000, Cell Signaling Technology, Danvers, MA, USA), and anti-rabbit IgG (H+L), F(ab')_2_ Fragment (Alexa Fluor^®^488 Conjugate) (1:1000, Cell Signaling Technology, Danvers, MA, USA) in TBS containing 5% goat serum and 0.1% Triton at room temperature for 1 h. The immunostaining was analyzed by using a laser scanning confocal microscope (Nikon, A1, Shanghai, China).

### Cell culture and RNA interference

A mixture of 50% neuroblastoma (N)2a and 50% BV2 cells was cultured in a humid incubator with 95% air and 5% CO_2_ at 37°C. The DMEM medium (Gibco, Grand Island, NY, United States) with 10% FBS (Gibco, Grand Island, NY, United States) was changed when the color changed to yellow. In one set of experiments, the cells were treated with the indicated concentration of KA (1, 5, 10, 20, or 40 μM) for 48 h before extracting total proteins. In another set of experiments, mixed cells were pre-transfected with or without siRNAs targeting Grik1 (200 ng, Santa Cruz Biotechnology, Shanghai, China) by using the FuGENE 5 transfection reagent (Roche Diagnostics, Indianapolis, IN, USA) according to the manufacturer’s instruction before stimulation with KA (10 μM) for 48 h. The control cells were treated with corresponding dilutions of DMSO.

In addition to the above experiments, the mixed cells were treated with KA (10 μM) only; topiramate (2 μM) (Sigma-Aldrich Corp, St. Louis, MO, USA), salubrinal (5 μM), SB216763 (2 μM), or Bay11-7082 (1 μM) (Sigma-Aldrich Corp, St. Louis, MO, USA) along with KA (10 μM); or vehicle (Control) and incubated for 48 h before total protein extraction was performed.

### Western blots

Total protein extraction was performed using a protein extraction kit (Thermo Fisher Scientific, Shanghai, China) following the manufacturer’s protocol. Protein extracts were dissolved in 10%–15% SDS-PAGE and then transferred to a PVDF membrane at 100 V for 1 h. After being blocked with 5% non-fat skimmed milk [diluted with Tris-buffered saline containing 0.1% Tween 20 (TBST)] for 1 h at room temperature, the membrane containing the protein extracts was incubated overnight with primary antibody (diluted with 2% bovine serum albumin in TBST) at 4°C. The following primary antibodies were used: anti-β-actin (1:5000, Cell Signaling Technology, Danvers, MA, USA); anti-p-NF-κB (1:2000, Cell Signaling Technology, Danvers, MA, USA); anti-NLRP3 (1:3000, Cell Signaling Technology, Danvers, MA, USA); anti-IL-1β (1:1000, Cell Signaling Technology, Danvers, MA, USA); anti-GSK3β (1:1000, Cell Signaling Technology, Danvers, MA, USA); anti-p-tau (1:1000, Cell Signaling Technology, Danvers, MA, USA); anti-tau (1:3000, Cell Signaling Technology, Danvers, MA, USA); anti-ATF6 (1:2000, Cell Signaling Technology, Danvers, MA, USA); anti-GRP78 (1:2000, Abcam, Cambridge, MA, USA), and anti-IRE1 (1:2000, Cell Signaling Technology, Danvers, MA, USA). On the second day, proteins were visualized using the enhanced chemiluminescence detection system (Thermo Fisher Scientific, Shanghai, China) after incubation with the corresponding secondary antibodies (1:10000, Cell Signaling Technology, Danvers, MA, USA) and visualized by Bio-Rad ChemiDocXRS devices (Bio-Rad Laboratories, Shanghai, China). For quantitative analysis of band intensity, we calculated the band intensity ratio for normalization by using Image J software. For each blot, the background subtracted density of the target protein in each lane was multiplied by the ratio of the density of the loading control (such as the housekeeping protein) from a control sample of all the study blots to the other lanes in the gel. This yielded the normalized density to the loading control (NDL). The fold difference for each replicate was calculated by dividing the NDL from each lane by the NDL from the control sample.

### Statistical analysis

Data were expressed as the mean ± standard deviation values and analyzed using SPSS 10.0 statistical software (SPSS Inc., Chicago, IL, United States). The one-way and two-way ANOVA tests were used to determine the significance of differences among groups (P < 0.05, P < 0.01, P < 0.001).

## Supplementary Material

Supplementary Figures
