# Supplementary material for: Kainic acid hyperphosphorylates tau via inflammasome activation in MAPT transgenic mice
Source: Aging (Albany NY). 2019 Dec 2;11(23):10923–38. doi: 10.18632/aging.102495 (PMC6932880; doi:10.18632/aging.102495)
Supplement: Supplementary Figures [file aging-11-102495-s001..pdf]

SUPPLEMENTARY FIGURES

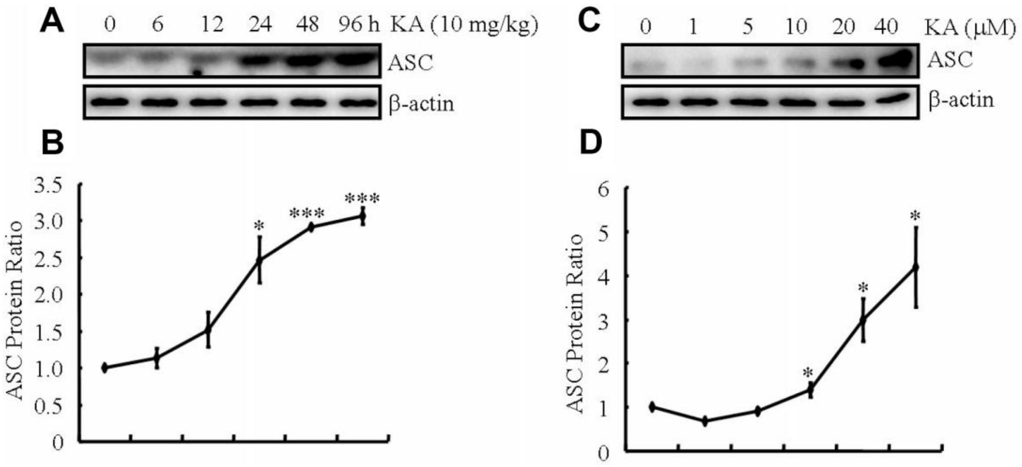

Supplemental Figure 1. KA induces the expression of ASC in vivo and in vitro. (**A**, **B**) The expression level of ASC in the KA-treated mouse brain at different time points. (**C**, **D**) The expression level of ASC in KA-treated mixed cells. The optical density of bands in western blots was analyzed by Image J software (**P* < 0.05, ****P* < 0.001 vs. controls; the significant differences from the respective values were determined by one-way analysis of variance test. N = 3 for western blotting).

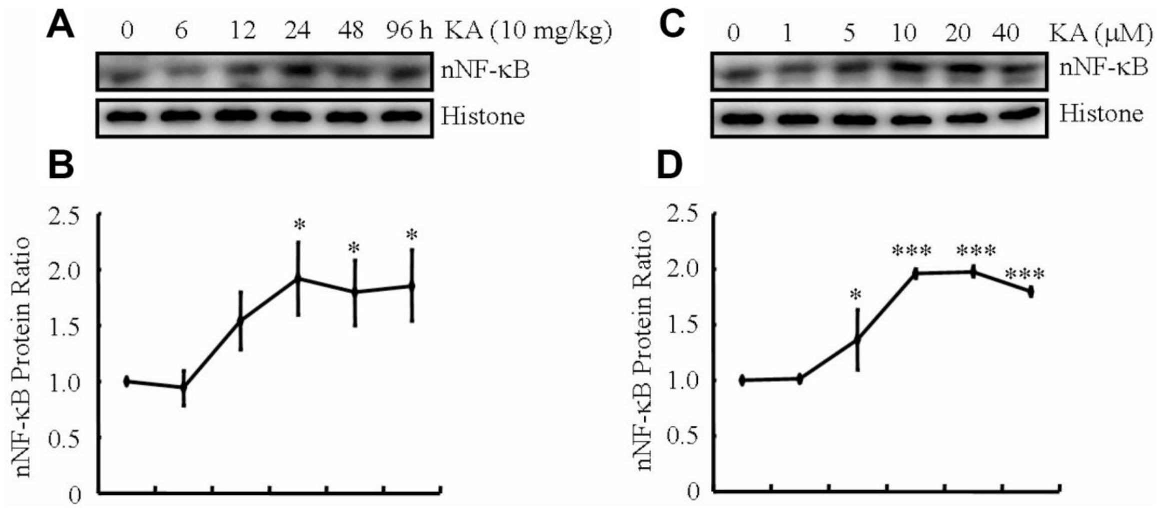

Supplementary Figure 2. KA increases the nucleus levels of NF-κB in vivo and in vitro. (**A**, **B**) The nucleus level of NF-κB in the KA-treated mouse brain at different time points. (**C**, **D**) The nucleus level of NF-κB in KA-treated mixed cells. The optical density of bands in western blots was analyzed by Image J software (**P* < 0.05, ****P* < 0.001 vs. controls; the significant differences from the respective values were determined by one-way analysis of variance test. N = 3 for western blotting).
